# Wirkung der multimodalen rheumatologischen Komplexbehandlung bei Patienten mit axialer Spondyloarthritis: Eine systematische Erhebung mit standardisierten Outcomeparametern wie dem ASAS-Gesundheitsindex

**DOI:** 10.1007/s00393-022-01241-1

**Published:** 2022-07-28

**Authors:** U. Kiltz, T. Wiatr, D. Kiefer, X. Baraliakos, J. Braun

**Affiliations:** 1 Rheumazentrum Ruhrgebiet, Claudiusstr. 45, 44649 Herne, Deutschland; 2 Ruhr Universität Bochum, Bochum, Deutschland

**Keywords:** Spondyloarthritis, Physikalische Therapie, Krankheitsaktivität, Funktionsfähigkeit, Lebensqualität, Spondylarthritis, Physical therapy, Disease activity, Functioning, Quality of life

## Abstract

**Hintergrund:**

Die multimodale rheumatologische Komplexbehandlung (MRKB) beruht auf einem akutstationären Versorgungskonzept zur Behandlung von Patienten mit klinisch relevanten Funktionseinschränkungen und Schmerzexazerbationen, die durch rheumatische und muskuloskeletale Erkrankungen bedingt sind. Patienten mit axialer Spondyloarthritis (axSpA) einschließlich der ankylosierenden Spondylitis (AS) leiden häufig unter solchen Gesundheitsproblemen. Regelmäßige Bewegungsübungen und physikalische Therapiemaßnahmen sind ein wichtiger Pfeiler im Behandlungsmanagement. Mit dem ASAS Health Index (ASAS HI) können die globale Funktionsfähigkeit und Gesundheit von axSpA-Patienten erfasst werden. Die Trennschärfe des ASAS HI für nichtpharmakologische Therapieänderungen ist bisher noch nicht nachgewiesen worden.

**Zielsetzung:**

Evaluation der im Rheumazentrum Ruhrgebiet durchgeführten MRKB und des ASAS HI für nichtpharmakologische Therapiemaßnahmen bei Patienten mit axSpA. Als primärer Endpunkt wurde eine Verbesserung des ASDAS ≥ 1,1 festgelegt. Hierbei wurde angenommen, dass > 25 % der Patienten diesen Schwellenwert erreichen.

**Methoden:**

Konsekutiv eingeschlossene Patienten mit aktiver axSpA und relevanten Funktionseinschränkungen wurden im Rahmen einer MRKB 14 Tage stationär behandelt. Alle Patienten beantworteten am ersten (V1) und am 14. Tag (V2) des Aufenthalts Fragebögen zu Schmerzen (NRS), Krankheitsaktivität (BASDAI, ASDAS) und Funktion (BASFI, ASAS HI). Die klinische Untersuchung erfolgte mittels BASMI und eine Messung des C‑reaktiven Proteins (CRP) zu beiden Zeitpunkten.

**Ergebnisse:**

Die prospektiv eingeschlossenen 66 Patienten hatten ein mittleres Alter von 47,2 Jahren (SD 14,2), eine Symptomdauer von ca. 20 Jahren, 65,3 % waren Männer und 75 % HLA B27+, das CRP war bei 41,3 % erhöht. Die Krankheitsaktivität zu V1 war erhöht: BASDAI 5,6 (1,8), ASDAS 3,1 (0,9), während Funktionsfähigkeit und Mobilität vermindert waren: BASFI 3,5 (1,8), BASMI 5,6 (2,1), ASAS HI 8,4 (3,4). Im Verlauf verbesserte sich das globale Patientenurteil (NRS 0–10) von 6,9 (1,7) zu V1 auf 4,8 (1,8) zu V2 und der Schmerz von 6,9 (1,9) auf 4,7 (2,0) (alle *p* < 0,001). Auch die Krankheitsaktivität nahm zu V2 ab: BASDAI 4,1 (1,9), ASDAS 2,4 (1,0), Funktion und Mobilität waren auch verbessert: BASFI 4,3 (2,4), BASMI 2,7 (1,6), ASAS HI 6,5 (3,8) (alle *p* < 0,001).

**Schlussfolgerung:**

In dieser Studie konnte die Wirksamkeit einer 2‑wöchigen MRKB gemäß OPS 8–983,1 hinsichtlich wichtiger patientenzentrierter Outcomes (PRO) nachgewiesen und frühere Studienergebnisse konnten bestätigt werden. In diesem Rahmen war auch der ASAS-HI veränderungssensitiv.

## Abstract (en)

**Background:**

Multimodal rheumatologic complex treatment (MRCT) is based on an acute inpatient treatment concept for patients with clinically relevant functional impairments and exacerbation of pain, which are caused by rheumatic and musculoskeletal diseases. Patients with axial spondylarthritis (axSpA) including ankylosing spondylarthritis (AS) often suffer from such health problems. Regular movement exercises and physical therapy measures are an important pillar of treatment management. The ASAS Health Index (ASAS-HI) can be used to document the global functional ability and health of axSpA patients. The selectivity of the ASAS HI for nonpharmacological treatment changes has so far not yet been proven.

**Objective:**

Evaluation of the MRCT and ASAS HI for nonpharmacological treatment measures of patients with axSpA carried out in the Ruhr Area Rheumatism Center. The primary endpoint was an improvement of the ASDAS≥ 1.1. It was assumed that > 25% of the patients would achieve this threshold.

**Methods:**

Consecutively included patients with active axSpA and relevant functional impairments received inpatient treatment for 14 days during MRCT. On days 1 (V1) and 14 (V2) all patients completed questionnaires on pain (NRS), disease activity (BASDAI, ASDAS) and function (BASFI, ASAS HI). The clinical examination was carried out using BASMI and measurement of C‑reactive protein (CRP) at both times.

**Results:**

The 66 prospectively included patients had an average age of 47.2 years (SD 14.2 years), a duration of symptoms of ca. 20 years, 65.3% were male, 75% were positive for HLA B27 and CRP was elevated in 41.3%. The disease activity at V1 was elevated: BASDAI 5.6 (1.8), ASDAS 3.1 (0.9), whereas functional ability and mobility were reduced: BASFI 3.5 (1.8), BASMI 5.6 (2.1), ASAS-HI 8.4 (3.4). During the course the global patient verdict improved (NRS 0–10) from 6.9 (1.7) at V1 to 4.8 (1.8) at V2 and the pain from 6.9 (1.9) to 4.7 (2.0) (all *p* < 0.001). The disease activity also decreased at V2: BASDAI 4.1 (1.9), ASDAS 2.4 (1.0), function and mobility were also improved: BASFI 4.3 (2.4), BASMI 2.7 (1.6), ASAS HI 6.5 (3.8) (all *p* < 0.001).

**Conclusion:**

In this study the effectiveness of a 2‑week MRCT according to OPS 8–983.1 with respect to important patient-centered outcomes (PCO) could be proven and the results of previous studies could be confirmed. In this context ASAS-HI was also sensitive to change.

Die axiale Spondyloarthritis (axSpA) ist durch Entzündungen und strukturelle Schäden an der Wirbelsäule gekennzeichnet, die zu Schmerzen, Funktionsverlust und Beeinträchtigung der sozialen Teilhabe führen können [1]. Eine frühzeitige Behandlung der Patienten mit axSpA ist notwendig, um das Ziel einer Remission zu erreichen und eine Verschlechterung der körperlichen Funktionsfähigkeit zu verhindern bzw. eine Verbesserung herbeizuführen [2, 3]. Die medikamentöse Behandlung umfasst neben nichtpharmakologischen Maßnahmen nichtsteroidale Antirheumatika (NSAR), konventionelle krankheitsmodifizierende Antirheumatika (csDMARDs) und Biologika (bDMARDs) [2]. Die Wirksamkeit der medikamentösen Therapie auf die Krankheitsaktivität und die körperliche Funktionsfähigkeit bzw. die Beweglichkeit der Wirbelsäule ist gut belegt [4, 5]. Parallel zu einer adäquaten medikamentösen Behandlung ist die Durchführung von regelmäßigen Bewegungsübungen, körperlicher Aktivität und auch gezielter Physiotherapie essenziell, um die Beweglichkeit der Wirbelsäule zu erhalten bzw. zu verbessern [2, 6]. Die multimodale rheumatologische Komplexbehandlung (MRKB, OPS 8–983) ist ein akutstationäres Versorgungskonzept (DRG I97Z) zur Behandlung akuter Funktionseinschränkungen und Schmerzexazerbationen, die durch rheumatische und muskuloskeletale Erkrankungen (nach EULAR RMD) bedingt sind. Hierunter fallen entzündlich rheumatische, aber auch degenerative und/oder weichteilrheumatische Erkrankungen wie die Fibromyalgie.

Der nach langen Jahren der Evaluierung 2015 veröffentlichte ASAS-Gesundheitsindex (Health Index [ASAS HI]) ist ein Messinstrument für die standardisierte Erfassung der globalen Funktionsfähigkeit und Gesundheit von Patienten mit axSpA [7]. Er wurde entwickelt, weil es kein valides Messinstrument zur Erfassung der globalen Funktionsfähigkeit und Gesundheit einschließlich des gesamten Symptom- und Behinderungskomplexes von Patienten mit axSpA gegeben hatte. Etablierte Messinstrumente erfassen nur einen Teil des gesamten Symptomkomplexes – wie z. B. Krankheitsaktivität und körperliche Funktionsfähigkeit [8, 9]. Die psychometrischen Charakteristika des ASAS HI sind in einer internationalen Validierungsstudie nachgewiesen worden [10]. Dabei zeigte sich, dass der ASAS HI valide und reliabel ist und zwischen verschiedenen Krankheitszuständen diskriminieren kann.

Während die Trennschärfe des ASAS HI für medizinische Interventionen nachgewiesen wurde, wurde sie für nichtpharmakologische Therapiemaßnahmen wie die physikalische Therapie bisher noch nicht nachgewiesen. Da regelmäßige Bewegungsübungen und physikalische Therapie ein wichtiger Teil der Behandlung von Patienten mit axSpA sind, ist es wichtig, die Diskriminationsfähigkeit und Änderungssensitivität des ASAS HI für nichtpharmakologische Behandlungen zu untersuchen.

Ziel dieser klinischen Studie war zum einen die Evaluation der MRKB in der klinischen Routine und zum anderen die Untersuchung der Änderungssensitivität des ASAS HI für nichtpharmakologische Therapiemaßnahmen.

## Methoden

### Patienten

Erwachsene Patienten mit der klinischen Diagnose einer axSpA, die die ASAS-Klassifikationskriterien für axSpA erfüllten [11], wurden zwischen 2016 und 2017 im Rheumazentrum Ruhrgebiet, Herne, konsekutiv in die Studie eingeschlossen, wenn sie eine erhöhte Krankheitsaktivität und klinisch relevante Funktionseinschränkungen aufwiesen und somit eine 14-tägige stationäre MRKB indiziert war [12]. Die MRKB ist ein akutstationäres Versorgungskonzept (DRG I97Z), für das im OPS 8–983 vom Deutschen Institut für Medizinische Dokumentation und Information (DIMDI). Mindestanforderungen definiert wurden [13]. Danach sind die Mindestmerkmale der MRKB: (I) Team unter fachärztlicher Behandlungsleitung (Facharzt für Innere Medizin mit dem Schwerpunkt Rheumatologie, Facharzt für Orthopädie und Unfallchirurgie mit der Zusatzweiterbildung Orthopädische Rheumatologie oder Facharzt für Orthopädie mit dem Schwerpunkt Rheumatologie), (II) Einsatz von mindestens 3 Therapiebereichen: Physiotherapie/physikalische Therapie, Ergotherapie, Schmerztherapie, kognitive Verhaltenstherapie, Gesprächspsychotherapie patientenbezogen in unterschiedlichen Kombinationen mit einer Therapiedichte von mindestens 11 h pro Woche und (III) prozessorientiertes Behandlungsmanagement mit standardisierter Befunderhebung, Bestimmung der Krankheitsaktivität, der Funktionseinschränkung und des Schmerzausmaßes zu Beginn und am Ende des stationären Aufenthaltes. Dabei müssen zur Beurteilung der Krankheitsintensität diagnosebezogen folgende Instrumente eingesetzt werden: Disease Activity Score 28 (DAS 28), Funktionsfragebogen Hannover, Bath Ankylosing Spondylitis Disease Activity Index (BASDAI) oder Bath Ankylosing Spondylitis Functional Index (BASFI). Zur Beurteilung der Schmerzintensität sind die numerische Rating-Skala/visuelle Analogskala (NRS/VAS) als Schmerzscore zu verwenden. Der unmittelbare Beginn der Schmerztherapie, Physiotherapie oder physikalischen Therapie muss gewährleistet sein. Unterschieden wird nach der Dauer der Behandlung zwischen 8–983,0 mit mindestens 7 bis höchstens 13 Behandlungstagen und 8–983,1 mit mindestens 14 bis höchstens 20 Behandlungstagen. Die in diese Untersuchung eingeschlossenen Patienten durchliefen im Rheumazentrum Ruhrgebiet eine auf 14 Tage festgelegte MRKB (OPS 8–983,1).

Zur Unterscheidung zwischen röntgenologischer axSpA (r-axSpA) bzw. ankylosierender Spondylitis (AS) und nichtröntgenologischer axSpA (nr-axSpA) wurden die ASAS-Klassifikationskriterien von 2009 [11] und die modifizierten New-York-Kriterien von 1984 verwendet [14].

### Datenerhebung

Studienspezifische Untersuchungen wurden am Aufnahmetag (V1) und am 14. Tag (V2) der MRKB durchgeführt.

#### Studienspezifische Untersuchungen an V1

Demografische und klinische Daten wie Alter, Geschlecht, Vorhandensein klinischer Symptome inklusive extraspinaler/extraartikulärer Manifestationen, Informationen zu Gelenkersatz- und Wirbelsäulenoperationen, Body Mass Index (BMI), Bildungs‑, Beschäftigungs- und Familienstand sowie aktuelle und vergangene Arbeitsunfähigkeitszeiten wurden bei allen Patienten erhoben. Die Befunde für HLA-B27 wurden aus der Routineversorgung übernommen, es handelte sich in der Regel um PCR-Tests.

#### Studienspezifische Untersuchungen an V1 und V2

##### Daten der Routineversorgung.

Daten zu Labor (CRP) und Bildgebung wurden aus dem Krankenhausinformationssystem übernommen. Magnetresonanz- (MR) und Röntgenbilder wurden mit dem Berlin Spine Score und dem Modified Stokes Ankylosing Spondylitis Spinal Score (mSASSS) ausgewertet. Die Bilder wurden von 2 unabhängigen Reviewern ausgewertet.

Die medikamentöse Therapie wurde kategorial (keine Therapie, NSAID-Monotherapie, DMARD-Monotherapie, Biologika-Monotherapie oder eine Kombinationstherapie) erhoben. Zur Bewertung der NSAR-Dosis wurde der ASAS NSAR-Score erhoben [15]. Der Wertehorizont des ASAS NSAR-Scores liegt zwischen 0 und 100, wobei ein Wert von 0 keine Einnahme und ein Wert von 100 die Maximaldosis pro Tag angibt.

#### Assessments

Der Arzt beantwortete die Globalfrage zum Gesamtgesundheitsstatus der Patienten, die mit einer NRS 0–10 und einer 4‑Punkte-Likert-Skala (sehr schlecht bis sehr gut) bewertet wurde. Die Mobilität der Wirbelsäule wurde mit dem BASMI gemessen [16].

Der Patient füllte folgende Fragebögen als Selbstauskunftsbogen aus: BASDAI [17], ASDAS [18], BASFI [19], ASAS HI [10], der EuroQol-Fünf-Dimensionen-Fragebogen (EQ-5D-5L und visuelle Analogskala 0–100 mm) [20] und der Short Form Survey 36-Item (SF-36) [21] und der Patient Acceptable Symptom State (PASS) [22]. Als Schwellenwert für eine gute, moderate und schlechte globale Funktionsfähigkeit wurden die validierten Schwellenwerte zur Beschreibung des ASAS HI-Funktionsstatus verwendet (gut < 5,0, moderat 5,0–11,99 und schlecht ≥ 12,0). [10]. Für den ASAS HI ist eine Differenz des ASAS HI-Summenscores von ≥ 3 Punkten als kleinste messbare Änderung berechnet worden („smallest detectable change“ [SDC]). Die Patienten beantworteten eine Globalfrage zu ihrem Gesamtgesundheitsstatus, die mit einer NRS 0–10 und einer 4‑Punkte-Likert-Skala (sehr schlecht bis sehr gut) bewertet wurde. Die Angaben zu Rückenschmerzen und nächtlichen Schmerzen wurden mit einem NRS von 0–10 erhoben.

### Statistik

Als primärer Endpunkt wurde eine ASDAS-Verbesserung ≥ 1,1 festgelegt wobei angenommen wurde, dass > 25 % der Patienten diesen Schwellenwert erreichen. Die Power-Kalkulation mit Annahmen eines Signifikanzniveaus von 0,025 bei Durchführung eines einseitigen Testverfahrens und einer Powerannahme von 0,9 ergab eine Mindestgruppengröße von 65 Patienten im Proportionstest für binominale Verteilung. Der Powerkalkulation lag die Hypothese zugrunde, dass ohne Intervention 10 % und mit Intervention 25 % eine ASDAS-Verbesserung ≥ 1,1 erreichen. Als sekundäre Endpunkte wurden die Veränderung des Arzt- und Patientenglobalurteils, Schmerz, CRP, BASDAI, BASMI, BASFI, ASAS HI, EQ-5D-Index und SF-36 definiert.

Die deskriptiven Daten werden als absolute Häufigkeiten und Prozentsätze dargestellt, wenn sie sich auf die qualitativen Variablen beziehen. Kontinuierliche Variablen werden als Mittelwert ± Standardabweichung (SD) ausgedrückt. Die Differenz der Variablen zu V1 und V2 wurde bei kontinuierlichen Variablen als Mittelwert ± SD und bei kategorialen Variablen als Prozentpunkte angegeben. Die Änderungssensitivität wurde als „standardised response mean“ (SRM) mit der Formel SRM = Differenz des ASAS HI Mittelwert/Standardabweichung der Differenz des ASAS HI-Mittelwerts berechnet. Das Ausmaß der Änderungssensitivität mit einer SRM von < 0,4 ist dabei als gering, von 0,4–0,79 als moderat und von ≥ 0,8 als groß zu beurteilen. Der t‑Test wurde verwendet, um kontinuierliche Variablen, und der McNemar-Test, um kategoriale Variablen zu einzelnen Zeitpunkten zu vergleichen. Ein *p*-Wert < 0,05 wurde als statistisch signifikant angesehen.

## Ergebnisse

Es wurden 66 axSpA-Patienten (26 [39,4 %] nr-axSpA, 40 [60,6 %] r‑axSpA) mit einem Durchschnittsalter von 47 Jahren prospektiv eingeschlossen. Etwa zwei Drittel waren männlich, und 75 % waren HLA-B27+. Weitere demografische Patientendaten sind in Tab. 1 aufgeführt. Die meisten Patienten hatte einen mittleren Schulabschluss (62 [86,1 %]), waren verheiratet bzw. in Partnerschaft lebend (45 [62,5 %]) und arbeiteten in Vollzeit (39 [54,2 %]). Insgesamt 29 von 57 erwerbstätigen Patienten (50,9 %) waren in den letzten 3 Monaten arbeitsunfähig.

**Table 1:** 

*Variable*^*a*^	*n* *=* *66*
Alter, Jahre	47,3 (13.5)
Männliches Geschlecht, *n* (%)	44 (66.7)
Symptomdauer, Jahre	21.1 (12.1)
Body Mass Index	28,1 (5,4)
Bambusstabwirbelsäule	5 (7,5)
Extraspinale Manifestationen^b^, *n* (%)	16 (242)
Extraartikuläre Manifestationen^c^, *n* (%)	27 (40.9)
*HLA-B27 positiv, n (%)*	*54 (81,8)*
mSASSS (*n* = 61)^d^	10,4 (17,6)
Berlin Score (*n* = 40)	4,9 (6,3)
Zustand nach Hüftgelenksersatz, *n* (%)	2 (3,0)
Zustand nach Wirbelsäulenoperation, *n* (%)	2 (3,0)

*HLA-B27* menschliches Leukozytenantigen-B27

^a^Alle Werte sind als Mittelwert (SD) angegeben, wenn nicht anders angegeben

^b^Zu den extraspinalen Manifestationen gehören Arthritis, Daktylitis und Enthesitis

^c^Zu den extraartikulären Manifestationen gehören Uveitis, Psoriasis und chronisch entzündliche Darmerkrankungen (CED)

^d^Der modifizierte Stoke Ankylosing Spondylitis Spine Score (mSASSS) und der Berlin Spine Score (BSS) wurde für Patienten mit adäquater Bildgebung berechnet

### Aufnahmeuntersuchung

Die Patienten hatten zu Beginn der Studie ein hohes Schmerzniveau, eine hohe Krankheitsaktivität, eine verminderte körperliche und globale Funktionsfähigkeit und ein eingeschränktes Bewegungsausmaß (Tab. 2). Drei Viertel der Patienten wiesen eine hohe bis sehr hohe Krankheitsaktivität auf (ASDAS ≥ 3,5 bei 21 [29,2 %] und ASDAS ≥ 2,1 bei 45 [62,5 %]). Die Entzündungsaktivität in der Wirbelsäule wurde bei 65 Patienten mittels MRT dokumentiert, wobei bei 26 Patienten (39,4 %) ein für SpA typisches Knochenmarködem detektiert wurde; 28 Patienten (42,4 %) hatten mindestens einen Syndesmophyten und wiesen einen moderat ausgeprägten Strukturschaden an der Wirbelsäule auf (mSASSS 10,4 [17,6]). Bei 5 Patienten lag eine Bambusstabwirbelsäule vor. Periphere Symptome lagen bei 16 (24,2 %) Patienten vor (Arthritis 7 [10,6 %], Daktylitis 1 [1,5 %], Enthesitis 8 [12,1 %]).

**Table 2:** 

Variable^a^	Einheit	V1	V2	Differenz V2 − V1	*p*-Wert
CRP, mg/dl	mg/dl	1,1 (1,5)	0,8 (1,3)	−0,2 (1,2)	< 0,001
Arztglobalurteil	NRS 0–10	5,9 (1,1)	3,2 (1,3)	−4,6 (1,3)	< 0,001
Arztglobalurteil, *n* (%)	Sehr gut	0 (0,0)	4 (5,6)	+4 (5,6)	< 0,001
	Gut	1 (1,4)	47 (65,3)	+46 (63,9)	
	Mittel	45 (62,5)	18 (25,0)	−27 (37,5)	
	Schlecht	25 (34,7)	2 (2,8)	−23 (31,9)	
	Sehr schlecht	1 (1,4)	1 (1,4)	0 (0)	
Patientenglobalurteil	NRS 0–10	6,9 (1,7)	4,8 (1,8)	−2,1 (2,1)	< 0,001
Patientenglobalurteil, *n* (%)	Sehr gut	0 (0,0)	4 (5,6)	+4 (5,6)	< 0,001
	Gut	2 (2,8)	23 (31,9)	+21 (29,1)	
	Mittel	23 (31,9)	32 (44,4)	+9 (12,5)	
	Schlecht	40 (55,6)	13 (18,1)	−27 (37,5)	
	Sehr schlecht	7 (9,7)	0 (0,0)	−7 (9,7)	
Schmerz	NRS 0–10	6,9 (1,9)	4,7 (2,0)	−2,1 (1,9)	< 0,001
Nächtliche Schmerzen	NRS 0–10	6,0 (2,5)	4,5 (2,4)	−1,5 (2,6)	< 0,001
BASDAI	0–10	5,6 (1,6)	4,1 (1,9)	−1,5 (1,6)	< 0,001
ASDAS	0–10	3,1 (0,9)	2,4 (1,0)	−0,7 (0,8)	< 0,001
ASDAS Status, *n* (%)	Inaktiv	0 (0,0)	9 (12,5)	+9 (12,5)	< 0,001
	Mäßig aktiv	6 (8,3)	19 (26,4)	+13 (18,1)	
	Aktiv	45 (62,5)	36 (50,0)	−9 (12,5)	
	Sehr aktiv	21 (29,2)	8 (11,1)	−13 (18,1)	
BASMI (*n* = 71)	0–10	3,5 (1,8)	2,7 (1,6)	−0,8 (0,7)	< 0,001
BASFI	0–10	5,6 (2,1)	4,3 (2,4)	−1,3 (1,5)	< 0,001
ASAS HI	0–17	8,4 (3,4)	6,5 (3,8)	−1,9 (2,7)	< 0,001
ASAS HI Status, *n* (%)	Gut	18 (25,0)	30 (41,7)	+12 (16,7)	< 0,001
	Mittel	40 (55,6)	33 (45,8)	−7 (9,8)	
	Schlecht	14 (19,4)	9 (12,5)	−5 (6,9)	
EQ-5D-Index^a^	0–1,0	0,5 (0,2)	0,7 (0,2)	0,2 (0,2)	< 0,001
EQ-5D allgemeine Skala	0–100	47,0 (20,3)	63,5 (18,8)	16,4 (18,2)	< 0,001
SF-36 körperliche Summenskala^a^	0–100	28,6 (7,0)	32,7 (8,2)	4,1 (5,6)	< 0,001
SF-36 psychische Summenskala^a^	0–100	45,0 (12,0)	46,5 (11,6)	1,4 (9,1)	< 0,001
Positive Patient Acceptable Symptom State, *n* (%)	–	13 (18,1)	46 (63,9)	+33 (45,8)	< 0,001

*NRS* Numerical Rating Scale, *ASAS HI* Assessment of SpondyloArthritis Health Index, *ASDAS* Ankylosing Spondylitis Disease Activity Score, *BASDAI* Bath Ankylosing Spondylitis Disease Activity Index, *BASFI* Bath Ankylosing Spondylitis Functional Index, *BASMI* Bath Ankylosing Spondylitis Metrology Index, *EQ-5D* EuroQol five dimensions questionnaire, *MRI* „magnetic resonance imaging“, *mSASSS* Modified Stoke Ankylosing Spondylitis Spinal Score, *SF-36* Short Form-36

^a^Werte sind als Mittelwert (SD) angegeben, wenn nicht anders angegeben

Die globale Funktionsfähigkeit wurde bei knapp 20 % der Patienten (14 [19,4 %]) als schlecht eingestuft, und 16 (22,2 %) der Patienten hatten depressive Symptome. Bei Aufnahme nahm die Mehrzahl der Patienten NSAR ein, überwiegend als Bedarfsmedikation, mit einem ASAS NSAR-Score von 49,9 (48,1). Knapp 20 % der Patienten nahmen bei Aufnahme bDMARDs. Knapp ein Viertel der Patienten erhielt bei Aufnahme csDMARDs und/oder Prednisolon.

### Multimodale Komplextherapie

Alle Patienten erfüllten die Mindestanforderungen für eine MRKB (OPS 8–983,1) und durchliefen ein auf axSpA-Patienten ausgerichtetes Programm: axSpA Gymnastik (45 min) und Bewegungsbad (30 min) täglich, Einzelkrankengymnastik und physikalische Therapie (Thermo- und Elektrotherapie sowie warmes Fango) (je 30 min 2‑ bis 3‑mal pro Woche), mindestens 3 Gruppentherapien (Fußgymnastik, Übungen mit Fitnessbänder, Tiefenmuskulaturtraining, Walking, Hockergymnastik oder Qigong jeweils 30 min), einmalig Rückenschule und Gelenkschutz (60 min) und Einweisung in die medizinische Gerätetherapie mit Trainingsplan zur Eigennutzung, sodass insgesamt mindestens 660 min Bewegungsangebote im Rahmen der MRKB/Woche durchgeführt wurden [12]. Darüber hinaus wurden psychologische Inhalte durch Gruppensitzungen zu den Themen Schmerz- und Krankheitsbewältigung und/oder Entspannungstherapie (jeweils 60 min) vermittelt.

Bei 11 (15,3 %) Patienten wurde während des stationären Aufenthaltes die Indikation zur Einleitung einer bDMARD-Therapie und bei 8 (11,1 %) die Indikation zur Einleitung einer csDMARD-Therapie gestellt.

### Folgeuntersuchung nach 2 Wochen

**Figure 1:**
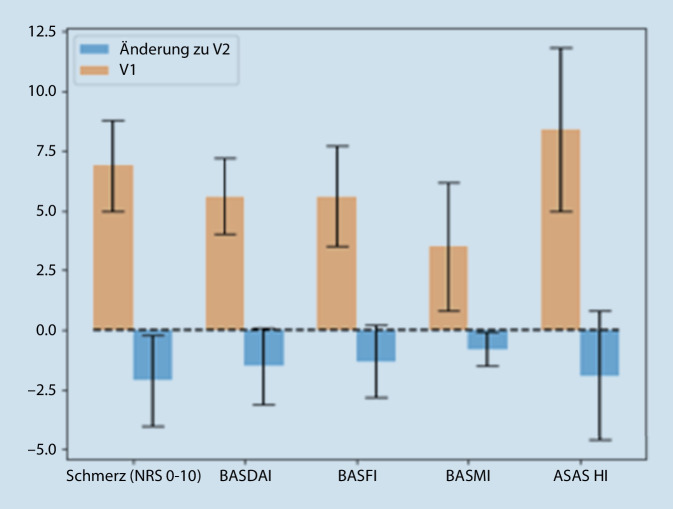

Alle Patienten beendeten die MRKB nach einer Liegedauer von 14 Tagen wie initial geplant. Die Patienten zeigten eine Verbesserung ihrer Schmerzen, der Krankheitsaktivität, der körperlichen und globalen Funktionsfähigkeit und der Beweglichkeit (Tab. 2, Abb. 1). Bei insgesamt 23 Patienten (31,9 %) verbesserte sich die Krankheitsaktivität um mindestens ≥ 1,1 Punkte im ASDAS. Nur noch 8 Patienten (11,1 %) wiesen eine hohe Krankheitsaktivität auf (ASDAS ≥ 3,5). Eine Verbesserung des ASAS HI von ≥ 3 Punkten (SDC) wurde bei 29 Patienten (40,3 %) dokumentiert. Hinsichtlich des ASAS-HI-Funktionsstatus gruppierten sich 4 Patienten weniger in die Kategorie „schlechte Funktionsfähigkeit“ ein, während 12 Patienten mehr von einer moderaten zu einer guten Funktionsfähigkeit wechselten.

Der ASAS HI wies nach 2‑wöchiger MRKB eine Änderungssensitivität von −0,70 auf und lag damit in einem moderaten Änderungsbereich.

## Diskussion

Die hier vorliegende Studie zeigt, dass die multimodale rheumatologische Komplextherapie bei Patienten mit axSpA auf verschiedenen Ebenen wirksam ist. Bedingt durch die intensive multimodale Therapie mit physiotherapeutischem Schwerpunkt in Kombination mit der im Rahmen des stationären Aufenthalts durchgeführten medikamentösen Therapie gelang es, sowohl die Krankheitsaktivität als auch die Funktionsfähigkeit und Beweglichkeit der axSpA-Patienten erheblich zu verbessern. Auch die Verbesserung der körperlichen Mobilität und Funktionsfähigkeit konnte in unserer Studie durch eine Verbesserung von BASMI und BASFI zu V2 objektiviert werden. Von besonderer Bedeutung ist, dass sich nicht nur die körperliche Funktionsfähigkeit, sondern auch die globale Funktionsfähigkeit verbessert hat. In der vorliegenden Untersuchung zeigte sich der ASAS HI somit änderungssensitiv im Rahmen der MRKB als nichtpharmakologisches Therapieverfahren. Das ist ein wichtiges Ergebnis, welches den Wert einer solchen intensiven Behandlung unterstreicht.

Die Effektivität der MRKB ist bei Patienten mit SpA kürzlich sowohl prospektiv als auch in einer retrospektiven Kohorte in Deutschland gezeigt worden [23, 24]. Dabei zeigten 59 prospektiv eingeschlossene SpA-Patienten (davon 32 mit axSpA) eine Verbesserung im Schmerzlevel von −1,5 (0,3), interessanterweise aber nicht hinsichtlich Krankheitsaktivität und körperlicher Funktionsfähigkeit. In der retrospektiven Analyse mit 134 Behandlungszyklen zeigte sich allerdings auch eine Verbesserung dieser wichtigen Parameter. Eine konsistente Verbesserung von Krankheitsaktivität und körperlicher Funktionsfähigkeit wurde auch in verschiedenen europäischen Studien gezeigt [25–29]. Allerdings können diese Interventionen nicht mit den Maßnahmen einer MRKB direkt verglichen werden. Es besteht keine Einigkeit darüber, wie lange die Verbesserungen einer MRKB insbesondere mit Blick auf die körperliche Funktionsfähigkeit erhalten werden können.

Für die hier vorliegende Studie als auch die oben erwähnten Untersuchungen aus Bad Nauheim ist allerdings kritisch anzumerken, dass eine Standardisierung der MRKB nicht gegeben ist und daher Therapieeffekte nicht kausal interpretiert werden können. Die MRKB der hier vorgestellten Studie wurde insofern standardisiert durchgeführt, als dass die Mindestanforderungen der OPS-Ziffer beachtet wurden. Eine MRKB kann im klinischen Alltag grundsätzlich aus verschiedenen Gründen nicht vollständig standardisiert werden – v. a. weil die Bedürfnisse der Patienten individualisiert betrachtet werden müssen. Zudem ist eine vollständige Standardisierung der MRKB in der klinischen Routine nicht möglich, weil es ständig Änderungsbedarf durch pragmatische Probleme wie Personalausfall durch Krankenstand etc. gibt.

Die globale Funktionsfähigkeit ist neben Krankheitsaktivität und körperlicher Funktion auch ein zentral wichtiger Outcomeparameter. Der ASAS-HI ist ein inzwischen sehr gut untersuchter Gesundheitsindex zur Erfassung der globalen Funktionsfähigkeit von Patienten mit axSpA. Die Änderungssensitivität für nichtpharmakologische Therapiemaßnahmen ist bisher nicht untersucht worden. Die hier vorliegende Studie zeigt diesbezüglich einen moderaten Effekt mit einer SRM von −0,7. Die Änderungssensitivität des ASAS HI für nichtpharmakologische Therapiemaßnahmen liegt damit auf einem vergleichbaren Niveau wie die Änderungssensitivität nach Einleitung einer csDMARD-Therapie (SRM von −0,69) und deutlich besser als nach Einleitung einer NSAR-Therapie (SRM von −0,44) [10]. Allerdings ist hierbei zu beachten, dass bei 15,3 % bzw. 11,1 % der Patienten eine bDMARD- bzw. csDMARD-Therapie erst während der MRKB eingeleitet wurde und damit eine Änderung auch im Bereich pharmakologischer Therapiemaßnahmen vorliegen konnte. Damit kann die hier berichtete Änderungssensitivität des ASAS HI nicht einfach und ausschließlich auf nichtpharmakologische Therapiemaßnahmen zurückgeführt werden. Allerdings ist ein relevanter Einfluss sowohl einer bDMARD- als auch einer csDMARD-Therapie bei einem Zeitintervall von 14 Tagen nicht unbedingt vorhanden, da bei beiden Wirkstoffgruppen die Wirksamkeit meist nicht unmittelbar einsetzt.

Der ASAS HI wurde inzwischen sogar als primärer Endpunkt in einer klinischen Strategiestudie („TICOSPA“) eingesetzt, und zwar als ≥ 30 % Verbesserung des Index [30]. Hierbei zeigte sich, dass sowohl eine Treat-to-target(T2T)-Strategie als auch eine Standardbehandlung auf hohem Niveau die globale Funktionsfähigkeit verbessern können. So fand sich eine solch 30 %ige Verbesserung der globalen Funktionsfähigkeit nach 1 Jahr Therapie bei 47,3 % der T2T- und bei 36,1 % der „normal“ behandelten Patienten. Bei dem in der TICOSPA-Studie verwendeten primären Outcome handelt es sich nicht um einen validierten Endpunkt, sodass dieser in dieser Studie nicht verwendet worden ist.

## Fazit

In der hier vorgelegten Studie gibt es zusammengefasst 2 berichtenswerte Ergebnisse: 1. Die stationär durchgeführte MRKB ist wirksam, was sich einerseits in patientenzentrierten Outcomes (PRO) niederschlägt als auch durch eine Besserung im Arztglobalurteil und Messungen der Wirbelsäulenbeweglichkeit sichtbar wird, und 2. der ASAS-HI ist ein valides Instrument für die Beurteilung der globalen Funktionsfähigkeit von Patienten mit axSpA, welcher auch unter der Bedingung einer nichtpharmakologischen Intervention sensitiv gegenüber Veränderung ist.
